# Risk factors and treatment outcomes of severe *Clostridioides difficile* infection in Singapore

**DOI:** 10.1038/s41598-019-49794-7

**Published:** 2019-09-17

**Authors:** H. L. Tay, A. Chow, T. M. Ng, D. C. Lye

**Affiliations:** 1 Department of Pharmacy, Tan Tock Seng Hospital, Singapore, Singapore; 2 Department of Clinical Epidemiology, Tan Tock Seng Hospital, Singapore, Singapore; 3 Department of Infectious diseases, Tan Tock Seng Hospital, Singapore, Singapore; 4 Lee Kong Chian School of Medicine, Nanyang Technological University, Singapore, Singapore; 5 Yong Loo Lin School of Medicine, National University of Singapore, Singapore, Singapore

**Keywords:** Bacterial infection, Clostridium difficile

## Abstract

Severe *Clostridioides difficile* infection (CDI) is associated with poorer outcomes. We aimed to identify risk factors and treatment outcomes of severe CDI. This was a retrospective cohort study. Eligible patients from January to December 2012 were recruited. Severity definitions were in accordance with SHEA/IDSA 2010 guideline. Treatment outcomes were (1) diarrhoea persistence, (2) CDI recurrence, (3) major complications despite treatment and (4) 30-day mortality. Two hundred and seventy-two patients were included and 40% had severe CDI. High APACHE II score (aOR 1.112, 95% CI 1.014–1.219; p < 0.05), high C-reactive protein (aOR 1.011; 95% CI 1.004–1.019; p < 0.01) and carbapenem usage in past 90 days (aOR 3.259; 95% CI 1.105–9.609; p < 0.05) were independent risk factors of severe CDI. Majority received oral metronidazole as sole treatment (92.6% for mild-moderate, 83.9% for severe, 77% for severe-complicated). Diarrhoea persistence was 32% versus 50% (p < 0.01), CDI recurrence 16.6% versus 16.5% (p > 0.05), major complications 1.2% versus 11% (p < 0.001) and 30-day mortality 7.4% versus 20.2% (p < 0.01) in mild-moderate CDI and severe CDI groups respectively. Oral metronidazole for severe CDI was associated with persistent diarrhoea, major complications and mortality. Risk factors for severe CDI can guide doctors in diagnosing severe CDI earlier and instituting oral vancomycin treatment to improve outcomes from severe CDI.

## Introduction

*Clostridioides difficile* infection (CDI) is the commonest cause of infectious diarrhoea in healthcare settings^1^. A worldwide concern, the number of CDI is rising and predicted to remain high^2^. In United States (U.S.), Steiner *et al*. reported more than double increase from 5.6 to 12.7 CDI per 1000 discharges from 2001 to 2011^3^. Such an alarming rate of increase has generated keen interest in CDI research for more than a decade.

In comparison to the trajectory of CDI research in the West, CDI research in Asia has been less robust hence limiting the knowledge on the prevalence, characterisation and the consequence of CDI in this region^4,5^. A recent systematic review on Asia demonstrated pooled incidence rate of 5.3 CDI per 10,000 patient days, numbers similar to the Europe and North America, suggesting that CDI in Asia should not be underestimated^6^. Furthermore, antibiotic use, an established risk factor of CDI, lacks strict regulations in many Asian countries especially in South East Asia where Singapore lies^7–9^. In Singapore, CDI incidence was between 5.16 to 6.64 per 10,000 patient days in 2006 and reportedly declined to 2.99 per 10,000 patient days in 2008^10,11^.

Since the Society for Healthcare Epidemiology of America (SHEA) and the Infectious Diseases Society of America (IDSA) updated the clinical practice guidelines for CDI in adults in 2010, the severity and treatment outcomes of CDI in Singapore remain unknown^12^. We aimed to describe CDI disease severity as defined by the SHEA/IDSA 2010 guideline, compliance with the international treatment guideline and associated disease outcomes. We also aimed to identify risk factors for severe CDI.

## Results

Three hundred forty-two unique patients had stool samples with positive C. *difficile* test from January 2012 to December 2012. Seventy patients were excluded; 35 patients had less than 3 stool counts when the sample was sent for C. *diffcile* testing, 10 patients had irretrievable case-notes, 12 had no documented hospital admission, 3 were on a stoma bag or rectal tube, 9 did not received any treatment despite a positive CDI result and 1 patient was a recurrent CDI case on treatment. Two hundred and seventy-two patients had positive C. *difficile* with documented diarrhoea and were included into the analysis. Baseline characteristics of the cohort are summarized in Table 1. Overall, patients who had severe forms of CDI appeared more ill compared to the mild-to-moderate CDI patients; median APACHE II scores and C-reactive protein value were significantly higher (p < 0.001) in the severe CDI group. Patients in the severe CDI group were also more likely to be in ICU unit, had lower albumin value and had concurrent infections as diagnosed by the attending physicians and were treated with other concurrent antibiotics.

**Table 1:** Baseline characteristics.

	Total (n = 272)	Mild-to-moderate (n = 163)	Severe and severe complicated (n = 109)	P value
Male, n (%)	116 (42.6)	64 (39.3)	52 (47.7)	0.168
Age, median year (IQR)	77 (67–84)	77 (68–84)	75 (66–82)	0.195
Age-adjusted Charlson comorbidity score, median (IQR)	6 (4–7)	6 (4–7)	6 (5–7)	0.468
APACHE II, median (IQR)	13 (9–17)	11 (8–16)	15 (11–20)	<0.001
Admitted for >48 hours prior to CDI, n (%)	225 (82.7)	135 (82.8)	90 (82.6)	0.957
On nasogastric tube prior to CDI, n (%)	92 (33.8)	55 (33.7)	37 (33.9)	0.972
Intensive care unit upon diagnosis, n (%)	19 (7.0)	4 (2.5)	15 (13.8)	<0.001
Prior antibiotic use in last 90 days, n (%)	258 (94.9)	154 (94.5)	104 (95.4)	0.733
Prior H2RA use in last 90 days, n (%)	60 (22.1)	34 (20.9)	26 (23.9)	0.559
Prior PPI use in last 90 days, n (%)	228 (83.8)	136 (83.4)	92 (84.4)	0.832
Albumin, median (IQR)	23 (20–27.5)^#^	24 (21–28)	22 (18–26)	0.029
C-reactive protein, median (IQR)	57.6 (25.6–138)*	43.2 (21–69.9)	116.50 (46.2–185.20)	<0.001
Concurrent H2RA use, n (%)	46 (16.9)	23 (14.1)	23 (21.1)	0.132
Concurrent PPI use, n (%)	202 (74.3)	119 (73.0)	83 (76.1)	0.562
Concurrent antibiotics use, n (%)	167 (61.4)	85 (52.1)	82 (75.2)	<0.001
Concurrent infection, n (%)	165 (60.7)	84 (51.5)	81 (74.3)	<0.001

^#^123 missing data *110 missing data.

IQR – Interquartile range, H2RA – Histamine-2 receptor antagonist, PPI – Proton pump inhibitors.

In the cohort, 163 (59.9%) had mild-to-moderate CDI, 56 (20.6%) had severe CDI and 53 (19.5%) had severe complicated CDI. Eleven patients (19.6%) with severe CDI had both leukocytosis and acute kidney injury. Thirty-five patients (62.5%) had leukocytosis of more than or equal to 15 while 10 patients (17.9%) presented with acute kidney injury. Among the 53 patients with severe complicated CDI, 50 patients (94%) had documented hypotension with 14 patients (26%) needing inotropic support for shock and 3 (6%) developed megacolon. None of the patients had documented ileus (0%).

### Risk factors for severe CDI

Variables associated with severe and severe complicated CDI included a higher APACHE II score, onset of CDI in the intensive care unit setting, a lower albumin level, higher C-reactive protein level and concurrent antibiotic use for treating infections other than CDI. Other variables included prior carbapenem usage in the past 90 days (p = 0.014) and concurrent respiratory tract infection with CDI (p = 0.037). Fitting these variables with Charlson’s comorbidity score into the multivariable logistic regression model, high APACHE II score (adjusted OR [aOR] 1.112, 95% CI 1.014–1.219; p = 0.024), high C-reactive protein (aOR 1.011; 95% CI 1.004–1.019; p = 0.004) and prior carbapenem usage in the past 90 days (aOR 3.259; 95% CI 1.105–9.609; p = 0.032) were identified as independent risk factors of severe and severe complicated CDI (Table 2)^13^.

**Table 2:** Factors associated with severe and severe, complicated CDI according to univariate analysis and multivariate logistic regression.

Variable	Univariate analysis		Multivariate analysis	
	OR (95% CI)	P value	aOR (95% CI)	P value
Age-adjusted Charlson comorbidity score	1.028 (0.940–1.124)	0.545	0.889 (0.710–1.113)	0.303
APACHE II	1.144 (1.091–1.200)	<0.001	1.112 (1.014–1.219)	0.024
Intensive care unit upon diagnosis	6.343 (2.045–19.676)	0.001	1.705 (0.229–12.694)	0.602
Albumin	0.936 (0.881–0.994)	0.032	1.022 (0.923–1.130)	0.679
C-reactive protein	1.013 (1.008–1.018)	<0.001	1.011 (1.004–1.019)	0.004
Prior use of carbapenems	1.928 (1.140–3.260)	0.014	3.259 (1.105–9.609)	0.032
Concurrent antibiotics use	2.787 (1.636–4.747)	<0.001	1.412 (0.442–4.513)	0.561
Concurrent respiratory infection	1.795 (1.034–3.116)	0.038	0.674 (0.202–2.249)	0.521

OR – Odds ratio, aOR – Adjusted odds ratio, CI – Confidence interval.

Subgroup analysis to assess any difference in the risk factors of severe CDI and severe complicated CDI was performed. Severe CDI was associated with a higher APACHE II score (p < 0.001), onset of CDI in the intensive care unit setting (p = 0.019), higher C-reactive protein level (p < 0.001) and concurrent antibiotic use for treating infections other than CDI (p = 0.003) compared with mild-to-moderate CDI but only high C-reactive protein was an independent risk factor of severe CDI (aOR 1.015; 95% CI 1.008–1.022; p < 0.001). Severe complicated CDI was associated with male gender (p = 0.028), higher APACHE II score (p < 0.001), onset of CDI in the intensive care unit setting (p = 0.001), higher C-reactive protein level (p = 0.001), prior carbapenem usage in the past 90 days (p = 0.036), concurrent H2RA use during CDI episode (p = 0.042) and concurrent antibiotic use for treating infections other than CDI (p = 0.004). High APACHE II score (aOR 1.132; 95% CI 1.029–1.246; p = 0.011), concurrent H2RA use (aOR 3.603; 95% CI 1.170–11.096; p = 0.026) and male gender (aOR 3.526; 95% CI 1.343–9.258; p = 0.010) were independent risk factors of severe complicated CDI.

### Treatment and outcomes

The overall guideline concordance for CDI treatment was 55.9%. Mild-moderate CDI had the highest guideline concordance while this was minimal for severe and severe complicated CDI group. Eighty-eight percent of all 272 episodes received only oral metronidazole. Of the 163 episodes of mild-moderate CDI, 92.6% received oral metronidazole, which is the recommended treatment. Majority of the patients received oral metronidazole 400 mg 8 hourly (n = 153). The median duration of oral metronidazole received was 12 days (interquartile range 10–15).

Of the 56 episodes of severe CDI, 83.9% were treated with oral metronidazole alone. Majority of the patients treated with oral metronidazole received 400 mg 8 hourly (n = 48). None of the patients in this group received the recommended treatment with oral vancomycin. Of the 53 episodes of severe complicated CDI, 77.3% were treated with oral metronidazole alone. All patients given oral metronidazole received 400 mg 8 hourly (n = 50). Only 1 patient received the recommended treatment of oral vancomycin with intravenous metronidazole. Other regimens prescribed for each severity category are stated in Table 3.

**Table 3:** CDI treatment regimens stratified by disease severity.

Mild-moderate CDI (n = 163)	n (%)
** PO metronidazole**	**151 (92**.**6)**
PO metronidazole followed by PO vancomycin	9 (5.5)
PO vancomycin followed by PO metronidazole	1 (0.6)
PO metronidazole + PO vancomycin concurrently	1 (0.6)
IV metronidazole	1 (0.6)
PO vancomycin	0 (0)
**Severe CDI (n** = **56)**	
PO metronidazole	47 (83.9)
PO metronidazole followed by PO vancomycin	6 (10.7)
PO vancomycin + IV metronidazole concurrently	1 (1.8)
PO metronidazole + PO vancomycin concurrently	1 (1.8)
IV metronidazole	1 (1.8)
** PO vancomycin**	**0 (0)**
**Severe**, **complicated CDI (n** = **53)**	
PO metronidazole	41 (77.3)
PO metronidazole followed by PO vancomycin	4 (7.5)
PO metronidazole + PO vancomycin concurrently	4 (7.5)
PO vancomycin followed by PO metronidazole	1 (1.9)
** PO vancomycin** + **IV metronidazole concurrently**	**1 (1**.**9)**
IV metronidazole	1 (1.9)
PO vancomycin	1 (1.9)

The overall rate of diarrhoea persistence despite treatment was 31.9% (52 of 163 patients) in the mild-to-moderate group and 50.5% (55 of 109 patients) in the severe and severe complicated CDI group (p = 0.002). Subgroup analysis showed that 41.1% (23 of 56 patients) of severe CDI (p = 0.212) and 60.4% (32 of 53 patients) of severe complicated CDI had diarrhoea persistence (p < 0.001).

Recurrence of CDI despite treatment was observed in 27 (16.6%) of 163 patients with mild-to-moderate CDI and in 18 (16.5%) of the remaining 109 patients (p = 0.991). Subgroup analysis showed that 10 (17.9%) of 56 severe CDI patients (p = 0.824) and 8 (15.1%) of 53 severe complicated CDI patients had recurrence (p = 0.801).

Major complications occurred in 1.2% (2 of 163 patients) in mild-to-moderate CDI and 11% (12 of 109 patients) in the rest of the patients (p < 0.001). Both patients with mild-to-moderate CDI developed septic shock despite treatment. For the remaining 12 patients, 8 developed septic shock despite treatment, 2 developed megacolon, 1 required colectomy and 1 developed septic shock requiring ICU admission and eventual colectomy. None of the patients developed intestinal perforation. Subgroup analysis showed that 8.9% (5 of 56 patients) of severe CDI (p = 0.013) and 13.2% (7 of 53 patients) of severe complicated CDI had major complications (p = 0.001).

Overall 30-day mortality was 12.5%. In mild-to-moderate CDI, 12 of 163 (7.4%) died while 22 of 109 (20.2%) remaining patients died within 30 days (p = 0.002). Subgroup analysis showed that 9 (16.1%) of 56 severe CDI patients (p = 0.056) and 13 (24.5%) of 53 severe complicated CDI patients died within 30 days from diagnosis (p = 0.001).

## Discussion

This is the first study in Singapore, to our best knowledge, to report on the severity of CDI and describe the treatment and outcomes of hospitalized patients. Approximately 40% of our CDI patients had severe and severe, complicated CDI. This figure is consistent with the proportion of severe CDI reported in some countries in the Asia region. Wong *et al*. reported 41.7% of severe CDI cases, with 16.5% being complicated in a prospective case-control study conducted in 3 major acute, general public hospitals in Hong Kong^14^. Their findings reflected closely to our findings as they too applied the SHEA/IDSA definition for severe CDI and the age of their CDI patients was similar to our population group (71.5 versus 77 years old). Another observational study in a tertiary hospital in Korea also reported 37.5% of severe CDI based on a severity scoring system^15^. Figures from these studies are comparable to studies in U.S. and Europe where the percentage of severe CDI ranged from 40–50%^16–18^. However, unlike U.S. and Europe, where the hypervirulent North American pulsed-field gel electrophoresis type 1 strain (NAP1/BI/027) have frequently been the cause of outbreaks and detrimental outcomes, ribotype 027 have been sporadically reported in Asia^5,19–22^. In Singapore, while there have been infrequent reports of ribotype 027, none were the hypervirulent strain^23–26^.

Our study population comprised mainly hospitalized, elderly patients with multiple comorbidities, with frequent prior exposure to healthcare facilities, antimicrobials and acid suppression especially proton-pump inhibitors. Independent risk factors of severe and severe complicated CDI in this study were high APACHE II score, high CRP and prior carbapenem usage. High CRP was the only independent risk factor of severe CDI while high APACHE II score, concurrent H2RA use and male gender were risk factors of severe complicated CDI. High APACHE II score reflected that the patients were sicker and more vulnerable to severe forms of CDI while high CRP was consistent with the severity of colitis. Elevated CRP as a predictor of severe CDI was reported in past studies^27–29^. Prior exposure to broad-spectrum antibiotics is also a known risk factor for CDI^14^. In a global point prevalence survey of antimicrobial usage in hospitals, Singapore hospitals had a high prevalence of >50% compared with other regions that ranged between 27–55%^30^. A multicentre study in Singapore indicated that 38% of carbapenem usage was inappropriate^31^. The high usage of antibiotics in Singapore hospitals is a potential modifiable risk that can be reversed. Few studies reported male gender as an association with severe CDI except a French study in medical ICU patients^27^. Age, co-morbidities and use of PPI were predictors for severe CDI in past studies not observed in this study^28,29^. While we did not find PPI exposure as a risk factor of severe CDI, acid suppression by H2RA remained a possible risk factor as it has been reported as a risk factor for CDI^32^.

An important finding of this study was the low usage of oral vancomycin to treat severe and severe complicated CDI. This may be due to the under-recognition of severe CDI as this is the first report in Singapore on the prevalence of severe CDI. The absence of hypervirulent CDI from a surveillance study^24^, concern of endemic vancomycin resistant enterococci^33^, and absence of affordable oral vancomycin tablets may also contribute to this observation. The concern of vancomycin-resistant enterococci with oral vancomycin is probably unfounded as both oral metronidazole and vancomycin exert similar effects^34^. Similar to some Europe and U.S. studies, the use of oral metronidazole for severe CDI cases was common and adherence to clinical practice guidelines for severe CDI treatment was reportedly poor in some Asia countries such as Korea and Japan^15,35^. In a tertiary hospital in Pittsburgh, 55% of patients had severe CDI but none received oral vancomycin^17^. In a French university hospital, 53.9% of CDI cases were identified as severe but only 1% of the patients received oral vancomycin while 25.5% received a combination of metronidazole and vancomycin^18^. In another tertiary care hospital in Houston, Texas, there were 41% of severe CDI cases but only 11% of all cases received oral vancomycin^16^. In Asia, Kim *et al*. reported 37.5% severe CDI cases but only 12.1% of the treated patients received vancomycin while Kobayashi *et al*. reported 37% severe CDI cases but only 18% of these cases adhered to the treatment recommended by SHEA/IDSA 2010 guideline for severe CDI^15,35^.

With regards to treatment outcomes, treatment failure with oral metronidazole and greater clinical success with oral vancomycin in severe CDI, were shown in two randomized controlled trials^36,37^. Zar *et al*. demonstrated that clinical cure, defined as resolution of diarrhoea by day 6 of treatment and a negative *C. difficile* toxin A assay, in patients with severe CDI was 76% when treated with oral metronidazole versus 97% when treated with vancomycin (p = 0.02)^37^. Similar to Zar *et al*., this study reflected 50.5% of diarrhoea persistence by day 6 in the severe CDI group where most were treated with oral metronidazole. This study also found no difference in recurrence of CDI despite severity. Our study findings of more persistent diarrhoea, major complications, and death with increasing severity of CDI predominantly treated with oral metronidazole supported experience from the literature that oral metronidazole is suboptimal for severe and severe complicated CDI^36–40^.

Our study had several limitations. Ours was a single-centred observational study conducted retrospectively. The prescribing habits observed in our institution may differ from other hospitals. Furthermore, our hospital serves mainly a geriatric population. The advanced age of patients in this study may have a negative impact on the treatment outcomes of CDI that may not be generalizable to a younger population. Additionally, this study was unable to demonstrate the impact of oral vancomycin since the number of CDI treated with oral vancomycin was very low. We used the SHEA/IDSA 2010 criteria for severe C difficile rather than the 2018 as this study was analysed before the release of the 2018 guideline; importantly the two criteria differ only in the definition of acute kidney injury with the 2018 requiring only serum creatinine above 1.5 mg/dL while the 2010 required a rise in serum creatinine >1.5 times that of baseline, which is more in line with the latest consensus on acute kidney injury KDIGO^41^. Lastly, the microbiology laboratory in our institution does not perform routine strain phenotyping, genotyping or ribotyping on its stool *C. difficile* samples. Hence, this study was unable to ascertain if treatment failure could be attributed to a more virulent or resistant strain of *C. difficile*.

## Conclusion

Our study documented significant burden of severe and severe complicated CDI in Singapore, the suboptimal treatment with oral metronidazole with persistent diarrhoea, major complications and mortality that correlated with severity of CDI. This emphasizes the importance of better surveillance for NAP1/BI/027 and increasing awareness among doctors of severe CDI and optimal treatment with oral vancomycin.

## Methods

### Study population

Tan Tock Seng Hospital (TTSH) is a 1500-bed acute care, university-teaching hospital in Singapore. From January 2012 to December 2012, all stool samples with a positive glutamate dehydrogenase (GDH) and positive *Clostridioides difficile* toxin A/B using enzyme immunoassay (EIA) method, Techlab *C. difficile* Quik Chek Complete, or indeterminate EIA test but positive polymerase chain reaction (PCR) using Xpert *C. difficile* PCR were extracted from our hospital’s microbiology database. Criteria for inclusion in the study were patients who: (1) were at least 18 years of age, (2) were hospitalized when the *C. difficile* diagnostic test was done and (3) had a positive *C. difficile* diagnostic test with 3 or more bowel movements a day documented within 24 hours of the positive diagnostic test. For patients who had more than 1 stool sample being tested for *C. difficile* within this time period, only the first positive sample was included. Exclusion criteria were patients who (1) were on stoma bag or rectal tube, (2) did not receive treatment, or (3) had irretrievable medical notes.

### Study design

This was a retrospective cohort study approved by the National Healthcare Group Domain Specific Review Board (NHG DSRB) which is our institutional review board (IRB) with waiver for informed consent – (Registration reference number 2013/00140). Demographics and clinical data were collected from our hospital electronic medical records and clinical case notes, and included age, gender, age-adjusted Charlson’s co-morbidity score^42^, APACHE II score^43^, ward location [intensive care unit (ICU) versus non-ICU] and clinical setting at onset (nosocomial or community). A history of antibiotic use and gastric acid suppressive agents [histamine-2 receptor antagonist (H2RA), proton pump inhibitor (PPI)] in the past 90 days prior to the onset of CDI was obtained. White blood cell count, C-reactive protein, procalcitonin, creatinine and albumin within 1 day of index stool sample were collected.

Definitions of mild-to-moderate, severe, and severe complicated CDI were in accordance with SHEA/IDSA 2010 guideline^12^. Severe CDI was defined as (1) leukocytosis with a white blood cell count of 15,000 cells/uL or higher, or (2) serum creatinine greater than or equal to 1.5 times the premorbid level. Severe complicated CDI was defined as the presence of (1) hypotension, (2) shock, (3) ileus or (4) megacolon.

### Treatment outcomes

The study treatment outcomes were (1) persistence of diarrhoea, (2) recurrence of CDI, (3) major complications despite treatment and (4) 30-day mortality from diagnosis. Diarrhoea was defined to have resolved if there were less than 3 unformed stools in 24 hours for 2 consecutive days. For patients who were discharged before the course of CDI treatment was completed, diarrhoea was taken to have resolved on the day of discharge. Persistent diarrhoea was defined as more than or equal to 3 unformed stools per day after at least 6 days of *C. difficile* treatment^37^. Recurrent CDI was defined as a positive *C. difficile* diagnostic test with 3 or more bowel movements a day within 2 to 8 weeks of the previous episode from the day of diagnosis^12^. Major complications included the need for admission into intensive care unit, development of septic shock, megacolon, intestinal perforation or colectomy despite treatment of CDI^12^.

### Statistical analysis

Chi-square test was used to compare categorical variables between groups. Fisher exact test was used when the expected counts were less than 5. Continuous variables were analysed using Mann-Whitney U test. Risk factors were determined by logistic regression fitted with variables that had p-values < 0.05 on univariate analysis and variables that are potential confounders based on literature review. For all statistical tests, a p-value of less than 0.05 was considered statistically significant. Association for the variables was reported as adjusted odds ratios (aORs) with a 95% confidence interval (CI). All statistical analyses were performed using IBM SPSS Statistics Version 21, 2012 (SPSS Inc.).
